# Optimal reference sequence selection for genome assembly using minimum description length principle

**DOI:** 10.1186/1687-4153-2012-18

**Published:** 2012-11-27

**Authors:** Bilal Wajid, Erchin Serpedin, Mohamed Nounou, Hazem Nounou

**Affiliations:** 1 Department of Electrical and Computer Engineering, Texas A&M University, College Station, TX 77843-3128, USA; 2 Department of Electrical Engineering, University of Engineering & Technology, Lahore, Punjab 54890, Pakistan; 3 Department of Chemical Engineering, Texas A&M University, Doha, Qatar; 4 Department of Electrical and Computer Engineering, , Doha, Qatar

## Abstract

Reference assisted assembly requires the use of a reference sequence, as a model, to assist in the assembly of the novel genome. The standard method for identifying the best reference sequence for the assembly of a novel genome aims at counting the number of reads that align to the reference sequence, and then choosing the reference sequence which has the highest number of reads aligning to it. This article explores the use of minimum description length (MDL) principle and its two variants, the two-part MDL and Sophisticated MDL, in identifying the optimal reference sequence for genome assembly. The article compares the MDL based proposed scheme with the standard method coming to the conclusion that “counting the number of reads of the novel genome present in the reference sequence” is not a sufficient condition. Therefore, the proposed MDL scheme includes within itself the standard method of “counting the number of reads that align to the reference sequence” and also moves forward towards looking at the model, the reference sequence, as well, in identifying the optimal reference sequence. The proposed MDL based scheme not only becomes the sufficient criterion for identifying the optimal reference sequence for genome assembly but also improves the reference sequence so that it becomes more suitable for the assembly of the novel genome.

## Introduction

Rissanen’s minimum description length (MDL) is an inference tool that learns regular features in the data by data compression. MDL uses “code-length” as a measure to identify the best model amongst a set of models. The model which compresses the data the most and presents the smallest code-length is considered the best model. MDL principle stems from Occam’s razor principle which states that “entities should not be multiplied beyond necessity”, http://www.cs.helsinki.fi/group/cosco/Teaching/Information/2009/lectures/lecture5a.pdf, stated otherwise, the simplest explanation is the best one, [1-5]. Therefore, MDL principle tries to find the simplest explanation (model) to the phenomenon (data).

The MDL principle has been used successfully in inferring the structure of gene regulatory networks [6-13], compression of DNA sequences [14-18], gene clustering [19-21], analysis of genes related to breast cancer [22-25] and transcription factor binding sites [26].

The article is organized as follows. Section 4 discusses briefly, the variants of MDL and their application to the comparative assembly. Section 4 explains the algorithm used for the purpose. Section 4 elaborates on the simulations carried out to test the proposed scheme. Section 4 explains the results and finally Section 4 points out the main features of this article.

## Methods

The relevance of MDL to Genome assembly can be realized by understanding that Genome assembly is an inference problem where the task at hand is to infer the novel genome from read data obtained from sequencing. Genome assembly is broadly divided into comparative assembly and de-novo assembly. In comparative assembly, all reads are aligned with a closely related reference sequence. The alignment process may allow one or more mismatches between each individual read and the reference sequence depending on the user. The alignment of all the reads creates a “Layout”, beyond which the reference sequence is not used any more. The layout helps in producing a consensus sequence, where each base in the sequence is identified by simple majority amongst the bases at that position or via some probabilistic approach. Therefore, this “Alignment-Layout-Consensus” paradigm is used by genome assemblers to infer the novel genome, [27-35].

Comparative assembly, therefore, is an inference problem which requires to identify a model that best describes the data. It begins the process by identifying a model, the “reference sequences”, most closely related to the set of reads. It then uses the set of reads to build on this model producing a model which overfits the data, the “novel genome”, [27,28,34,36-41]. The task of MDL is to identify the model that best describes the data and within comparative assembly framework the same meaning applies to finding the reference sequences that best describes the set of reads.

MDL presents three variants Two-Part MDL, Sophisticated MDL and MiniMax Regret [1]. The application of these will be briefly discussed in what follows.

### Two-part MDL

Also called old-style MDL, the two-part MDL chooses the hypothesis which minimizes the sum of two components: 

•The code-length of the hypothesis.

•Code-length of the data given the hypothesis.

The two-part MDL selects the hypothesis which minimizes the sum of the code-length of the hypothesis and code-length of the data given the hypothesis, [1,42-47]. The two-part MDL fits perfectly to the comparative assembly problem. The potential hypothesis which is closely related to the data, in comparative assembly, happens to be the reference sequence whereas the data itself happens to be the read data obtained from the sequencing schemes.

### Sophisticated MDL

The two components of the two-part MDL can be further divided into three components:

•Encoding the model class: *l*(*M*_*i*_), where *M*_*i*_ belongs in model class, and *l*(*M*_*i*_) denotes the length of the model class in bits.

•Encoding the parameters (*θ*) for any model *M*_*i*_ : *l*_*i*_(*θ*).

•Code-length of the data given the hypothesis is $\text{lo}g_{2}\frac{1}{p_{\bar{\theta }}(X)}$.

where $p_{\bar{\theta }}(X)$ denotes the distribution of the Data X according to the model $\bar{\theta }$. The three part code-length assessment process again can be converted into a two-part code-length assessment by combining steps B and C into a single step B.

•Encoding the model class: *l*(*M*_*i*_), where *M*_*i*_ belongs to any Model class.

•Code-length of the Data given the hypothesis class $(M_{i})=l_{\left(M_{i}(X)\right)}$, where X stands for any data set.

Item (B) above, i.e., the ‘length of the encoded data given the hypothesis’ is also called the “stochastic complexity” of the model. Furthermore, if the data is fixed, or if item (B) is constant, then the job reduces to minimizing *l*(*M*_*i*_), otherwise, reducing part (A), [1,48-53].

### MiniMax regret

MiniMax Regret relies on the minimization of the worst case regret, [49,50,53-59]:

(1) $$\underset{M}{\text{min}}\underset{X}{\text{max}}\left[\text{loss}(M,X)-\underset{\hat{M}}{\text{min}}\text{loss}(\hat{M},X)\right],$$

where *M* can be any model, $\hat{M}$ represents the best model in the class of all models and X denotes the data. The Regret, $R_{M_{i},X}$, is defined as

(2) $$R_{M_{i},X}=\left[\text{loss}(M_{i},X)-\underset{\hat{M}}{\text{min}}\text{loss}(\hat{M},X)\right]$$

Here the loss function, $\text{loss}(M_{i},X)$, could be defined as the code-length of the data X, given the model class *M*_*i*_. The application of Sophisticated MDL in the framework of comparative assembly will be discussed in what follows.

### Sophisticated MDL and genome assembly

In reference assisted assembly, also known as comparative assembly, a reference sequence is used to assemble a novel genome from a set of reads. Therefore, the best model is the reference sequence most closely related to the novel genome and the data at hand are the set of reads.

However, it should be pointed out that the aim is not to find a general model, rather, the aim is to find a “model that best overfits the data” since there is just one or maybe two instances of the data, based on how many runs of the experiment took place. One “run” is a technical term specifying that the genome was sequenced once and the data was obtained. The term “model that best overfits the data” can be explained using the following example.

Assume one has three Reads {X, Y, and Z} each having *n* number of bases. Say reference sequences (L) and (M), where (L) = XXYYZZ and (M) =XYZ contains all three reads placed side by side. Since both models contain all the three reads, the stochastic complexity of both (L) and (M) is the same and both overfit the data perfectly. However, since (M) is shorter than (L), therefore (M) is the model of choice on account of being the model that “best” overfits the data.

To formalize the MDL process, the first step would be to identify the following considerations: 

•Encoding the model class: *l*(*M*_*i*_), *M*_*i*_ belongs to Model classes.

•Encoding the parameters (*θ*) of the Model *M*_*i*_ : *l*_*i*_(*θ*).

•Code-length of the data given the hypothesis is $\text{lo}g_{2}\frac{1}{p_{\bar{\theta }}(D)}$.

The model class in comparative assembly would be the reference (Ref.) sequence itself. The parameters of the model *θ*, are such that, *θ* ∈ {−1, 0, 1}. In the process of encoding the model class regions of the genome that are covered by the reads of the unassembled genome are flagged with “1”(s). Areas of the Ref. genome not covered by the reads are flagged as “0”(s), whereas areas of the Ref. genome that are inverted in the novel genome are marked with “−1”(s). In the end, every base of the Ref. sequence is flagged with {−1, 0, 1}. Therefore, the code-length of the parameters of the model is proportional to length of the sequence.

Data given the hypothesis is typically defined as “Number of reads that align to the Ref. sequence”. In the case presented below “data given the hypothesis” is defined in an inverted fashion as the “Number of reads that do not align to the reference sequence”. These two are interchangeable as the “Total number of reads” is the sum total of the “number of reads that aligned to the Ref.” and the “number of reads that do not align to the Ref.”.

Table 1 shows that choosing the reference sequence having the highest number of reads present is not a sufficient condition for selecting the optimal reference sequence. The simulation carried out compared two reference sequences Fibrobacter succinogenes S85 (NC_013410.1), [60,61], and Human Chromosome 21 (AC_000044.1), [62-64], with the reads of Pseudomonas aeruginosa PAb1 (SRX000424), [48,65,66]. It shows that in order to choose the optimal reference sequence one has to take into account both the “Code-length of the model” and “Number of reads found” to be the sufficient conditions for choosing the optimal reference sequence.

**Table 1:** Counting number of reads not enough

**S.No.**	**Reference sequence**	**Number of bases in genomes**	**Number of reads found**
1	Fibrobacter succinogenes subsp. succinogenes S85 (NC_013410.1)	3842635	157
2	Human Chromosome 21 (AC_000044.1)	32992206	158

The table shows that choosing the reference sequence which has the highest number of reads present is not a sufficient condition. Just by looking at the “Data given the model” ≡“Number of reads found” one ends up choosing Human Chromosome 21. However, looking at the fact that Chromosome 21 is about 9×larger than S85 one realizes that actually S85 is the model of choice. Furthermore, S85 is a bacterial genome whereas Chromosome 21 comes from a eukaryote genome. PAb1 is also a bacteria, therefore, S85 is most definitely the model of choice.

**Figure 1:**
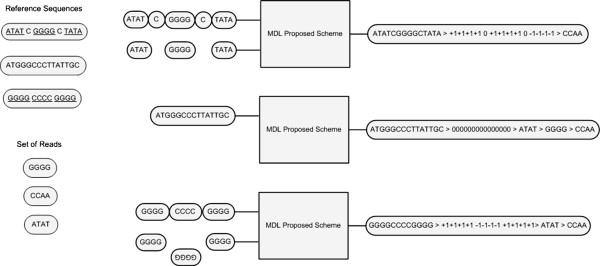
**MDL proposed scheme: The output of the system shows that the three components of the encoding scheme are separated from one another by “>”.** The scheme follows the format “Model > Model given the Data > Data given the hypothesis”. In the genome assembly framework the scheme mentioned above translates into “Reference Sequence >Reference Sequence according to the set of reads > Set of reads according to the Reference sequence”. “Model given the Data” is identified using {−1, 0, 1}. “1”(s) represent the base locations where the reads are found. “0”(s) represents the locations which are not covered by any read. “−1”(s) represents the locations of the genome that are inverted.

**Figure 2:**
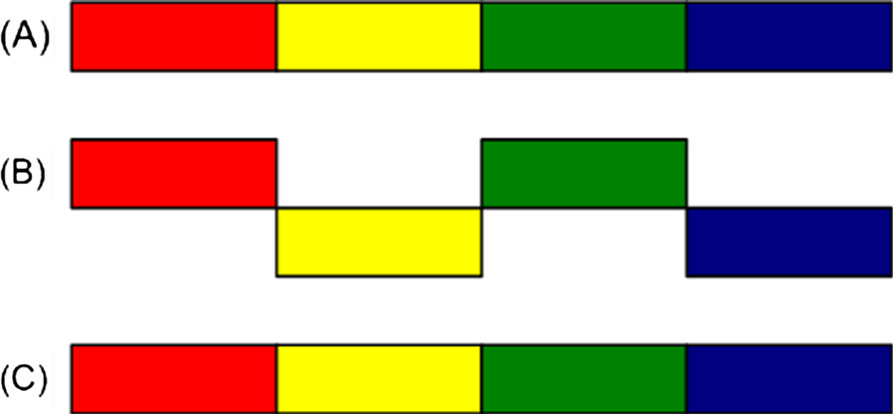
**Correcting inversions in the reference sequence.(a)** Reads are derived from the novel sequence. **(b)** The reference sequence, *S*_*R*_, contains two inversions, shown as yellow and blue regions. **(c)** The sequence generated *θ*has both yellow and blue regions rectified. Notice that using a simple ad-hoc scheme of counting the number of reads in the reference sequence one would have made use of **(b)** for assembly of novel genome. However, using MDL one can now use **(c)** for the assembly of the novel genome.

**Figure 3:**
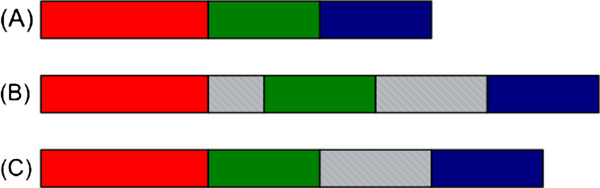
**Removing insertions in the reference sequence.(a)** Reads are derived from the novel sequence. **(b)** The reference sequence, *S*_*R*_, contains two insertions, shown as shaded grey boxes. **(c)** The proposed MDL process generates *θ*. The process removes only those insertions which are larger than *τ*_1_ but smaller than *τ*_2_; where *τ*_1_ and *τ*_2_ are user-defined. To remove the other insertion the value of *τ*_2_ could be increased.

Therefore, a simple yet novel scheme is proposed for the solution to the problem, see Figure 1 and Table 2. The proposed scheme follows the three assessment process of Sophisticated MDL. The MDL based proposed scheme stores the model class (Ref. sequence), the parameters of the model (where each base of the sequence is flagged with {−1, 0, 1}) and the data given the hypothesis (reads of the novel genome that do not align to the Ref. sequence) is one file. The file is than encoded using either Huffman Coding [67-70] or Shannon-Fano coding [68-71] to determine the code-length. For a simplistic three bits per character coding the code-length is measured according to Equation (3). The proposed scheme not only allows to determine the best model, amongst the pool of models to choose from, but also improves the model to be better suited according to the novel genome to be assembled. This is done by identifying all insertions and inversions, larger than one read length. It then removes those insertions and rectifies those inversions to get a better model, better suited to assemble the novel genome compared to what was started from, see Figures 2 and 3.

(3) $$\begin{array}{lll} \text{Code length}\;=\; & ({\text{Length}}_{\text{Ref. Seq.}}\times 3)\; & \;\\ & +({\text{Length}}_{\text{Parameters of the Model}}\times 3)\; & \;\\ & +({\text{Length}}_{\text{Read}}\times 3\times \text{No. of Unique}\; & \;\\ & \;\;\text{Unaligned Reads}).\; \end{array}$$

**Table 2:** Summary of the experiment using three reads {ATAT, GGGG, CCAA} and three reference sequences {1, 2, 3}

			**Reads that do not align to the reference sequence**	**Data given the hypothesis (Bits)**			**Code-length (Bits)**
		**Model given by the Data**					**Code-length**
**S.No.**	**Ref. Seq.**				***Regret***	**Proposed scheme**	**(Bits)**
1	ATATCGGGGCTATA	1111011110-1-1-1-1	CCAA	12	0	ATATCGGGGCATAT>1111 0 1111 0 -1-1-1-1>CCAA	102
2	ATGGGCCCTTATTGC	000000000000000	ATAT>GGGG>CCAA	42	30	ATGGGCCCTTATTGC> 000000000000000 >ATAT>GGGG >CCAA	138
3	GGGGCCCCGGGG	1111-1-1-1-11111	ATAT>CCAA	27	15	GGGGCCCCGGGG>1111-1-1-1-11111>ATAT>CCAA	105

Regret is defined as $R_{M_{i},X}=\left[\text{loss}(M_{i},X)-\underset{\hat{M}}{\text{min}}\text{loss}(\hat{M},X)\right]$. Here the loss function, $\text{loss}(M_{i},X)$, happens to be code-length of the data X, given the model class *M*_*i*_. Whereas, “Data given the hypothesis”, is the code-length of the “Reads that do not align to the reference sequence”. The code-length in the last column is measured according to Equation (3). The experiment shows that given the MDL proposed scheme Ref. 1 is the optimal choice for a reference sequence.

## 

### Algorithm 1 MDL Analysis of a Ref. sequence given aset of reads of the unassembled genome

## MDL algorithm

The pseudo code for analysis using sophisticated MDL and the scheme proposed in Section 4 is shown in Algorithm 1. Given the reference sequence *S*_*R*_ and *K* set of reads, {*r*_1_,*r*_2_,…,*r*_*K*_} ∈ *R*, obtained from the FASTQ [72,73] file, the first step in the inference process is to filter all low quality reads. Lines 3–10 filters all the reads that contain the base *N* in them and also the reads which are of low quality leaving behind a set of *O* reads to be used for further analysis. This pre-processing step is common to all assemblers. Once all the low quality reads are filtered out, the remaining set of *O* reads are sorted and then collapsed so that only unique reads remain.

Lines 13–27 describe the implementation of the proposed scheme as defined in Section 4. Assume that *S*_*R*_ is *l* bases long, and the length of each read is *p*. Therefore, $\phi_{S_{R}}$ picks up *p* bases at a time from *S*_*R*_ and checks whether or not $\phi_{S_{R}}$ is present in the set of collapsed reads *R*^′^. In the event $\phi_{S_{R}}\in R^{\prime }$ then the corresponding location on *S*_*R*_, i.e., *j* → *j* + *p* are flagged with “1(s)”. If $\phi_{S_{R}}\notin R^{\prime }$, then invert $\phi_{S_{R}}\to \psi_{S_{R}}$ and check whether or not $\psi_{S_{R}}\in R^{\prime }$. If yes, then mark the corresponding location on *S*_*R*_, i.e., *j* → *j* + *p* with “−1(s)” and flag $\phi_{S_{R}}$ to be present in *R*^′^. Otherwise, mark the corresponding locations on *S*_*R*_ as “0(s)”.

Lines 28–34 generates a modified sequence *θ*which has all the inversions rectified in the original sequence *S*_*R*_. Lines 35–44 identifies all insertions larger than *τ*_1_ and smaller than *τ*_2_ and removes them, see Figure 3. Here *τ*_1_ and *τ*_2_ are user-defined. Care should be taken to avoid removing very large insertions as this may affect the overall performance in deciding the best sequence for genome assembly. Lines 45–47 removes all the reads that are present in the original *S*_*R*_ and the modified sequence *θ* identified by flags 1 and −1. In the end the code-lengths are identified by any popular encoding scheme like Huffman [67-70] or Shannon-Fano coding [68-71]. If *ξ* is the smallest code-length amongst all models then use *θ*as a reference for the assembly of the unassembled genome rather than using *S*_*R*_.

## Results

Simulations were carried out on both synthetic data as well as real data. At first, the MDL process was analyzed on synthetic data on four different sets of mutations by varying the number and length of {Single nucleotide polymorphisms (SNPs), Inversions, Insertions, and Deletions}. The experiments using synthetic data were carried out by generating a sequence *S*_*N*_. The set of reads were derived from *S*_*N*_ and sorted using quick sort algorithm [74,75]. Each experiment modified *S*_*N*_ to produce two reference sequences *S*_*R*1_ and *S*_*R*2_ by randomly putting in the four set of mutations. The choice of the best reference sequence was determined by the code-length generated by the MDL process. See Tables 3, 4, 5, and 6 for results.

**Table 3:** Variable number of SNPs: the experiment shows the effect of increasing the number of SNPs on choice of the reference sequence

**Ref. Seq.**	**SNPs**	**No. of inversions**	**No. of insertions**	**No. of deletions**	**Code-length using proposed scheme (Kb)**
1	183	52 / 52	62 / 59	62	1815.14
2	224	50 / 51	66 / 58	63	1843.35

*S*_*R*2_ has higher number of SNPs as opposed to *S*_*R*1_. The code-length suggests that *S*_*R*1_ is the model of choice as it has a smaller code-length. The results show that the MDL scheme works successfully on variable number of SNPs by choosing the model with a lower number of SNPs in them.

**Table 4:** Variable number of insertions: the experiment shows the effect of increasing the number of insertions on choice of the reference sequence

**Ref. Seq.**	**SNPs**	**No. of inversions**	**No. of insertions**	**No. of deletions**	**Code-length using proposed scheme (Kb)**
1	0	0	136 / 196	0	1200.3
2	0	0	132 / 203	0	1228.25

The location and length of these insertions was chosen randomly. $\frac{136}{196}$ shows that out of 196 insertions in *S*_*R*1_ only 136 were removed. The remaining insertions were not recovered due to the choice of *τ*_1_ and *τ*_2_. *S*_*R*2_ has higher number of insertions as opposed to *S*_*R*1_. The code-length suggests that *S*_*R*1_is the model of choice as it has a smaller code-length.

**Table 5:** Variable number of deletions: the experiment shows the effect of increasing the number of deletions on choice of the reference sequence

**Ref. Seq.**	**SNPs**	**No. of inversions**	**No. of insertions**	**No. of deletions**	**Code-length using proposed scheme (Kb)**
1	0	0	2 / 0	182	1997.28
2	0	0	3 / 0	189	2015.35

The location and length of these deletions was chosen randomly. *S*_*R*2_ has higher number of deletions as opposed to *S*_*R*1_. The code-length suggests that *S*_*R*1_ is the model of choice as it has a smaller code-length. The experiment show that although no insertions were put in the actual sequence yet still two and three insertions were found for *S*_*R*1_and *S*_*R*2_, respectively. This may be due to a large section of reads that could not align to the reference sequence on the edges of these deletions.

**Table 6:** Variable number of inversions: the experiment shows the proposed scheme is robust to the number of inversions in the reference sequence

**Ref. Seq.**	**SNPs**	**No. of inversions**	**No. of insertions**	**No. of deletions**	**Code-length using proposed scheme (Kb)**
1	0	0	0	0	586.04
2	0	176 / 176	0	0	586.04

Both *S*_*R*1_ and *S*_*R*2_ have the same code-length. This is because the MDL scheme not only detected all the inversions for *S*_*R*2_ but also recovered all of them. So effectively *S*_*R*2_ ≡ *S*_*R*1_ after the MDL process as explained in Figure 2.

Once the robustness of MDL scheme on each of the four types of mutations was confirmed two-set of experiments were carried out on real data using Influenza viruses A, B, and C which belong to the Orthomyxoviridae group. Influenza virus A has five different strains, i.e., {H1N1, H5N1, H2N2, H3N2, H9N2}, while Influenza viruses B and C each have just one. The genomes of Influenza viruses is divided into a number of segments. Influenza virus A and B each have eight segments while virus C has seven segments, [76-78]. Amongst the first segments of each of the viruses only one was randomly selected and then modified to be our novel genome, *S*_*N*_. Reads were then derived from *S*_*N*_ and compared with all the seven reference sequences. See Table 7 for results.

**Table 7:** Simulations with Influenza virus A, B, and C

**S.No.**	**Ref. Seq. (Influenza virus)**	**No. of inversions**	**No. of deletions**	**Code-length using proposed scheme (Kb)**
1	A, H1N1 (NC_002023.1)	0 / 4	1	254.109
2	A, H5N1 (NC_007357.1)	0 / 4	1	254.109
3	A, H2N2 (NC_007378.1)	0 / 4	1	254.109
4	A, H3N2 (NC_007373.1)	0 / 4	1	254.109
5	A, H9N2 (NC_004910.1)	0 / 4	1	254.109
6	B (NC_002204.1)	4 / 4	1	68.62
7	C (NC_006307.1)	0 / 4	1	254.027

One of the sequences from Influenza virus {A, B, C} was randomly selected and modified to include {SNPs =7, inversions =4, deletions =1, insertions =3}. As Influenza virus A has five different strains while both Influenza viruses B and C each have one the MDL process was used to compare the seven sequences to determine which is the best reference sequence. Ref. Seq. 6, Influenza virus B was found to have the smallest code-length (68.62Kb), and is therefore, the model of choice. The experiment also shows that given the optimal reference sequence, in this case Influenza virus B, the MDL process rectifies all inversions (4/4). However, given non-optimal reference sequences, the proposed MDL process is not able to rectify the inversions (0/4). So the proposed algorithm chooses the optimal reference sequence, and given the optimal reference sequence if not all, at least most of the inversions are also corrected.

The second-set of experiments analyzed the performance of the MDL proposed scheme on reference sequences of various lengths. The test was designed to check whether the proposed scheme chooses smaller reference sequence with more number of unaligned reads or does it choose the optimal reference sequence for assembly. The reads were derived from Influenza A virus (A Puerto Rico 834 (H1N1)) segment 1. All the reference sequences used in this test were also derived from the same H1N1 virus, however, with different lengths, see Tables 8 and 9.

**Table 8:** The experiment uses the proposed MDL scheme on the same set of reads but different set of reference sequences

**S.No.**	**Ref. Seq. (%)**	**No. of unaligned reads**	**Code-length (KB)**	**Execution time (s)**	**Length of new Seq.**
1	1	696	128.60	0.046	14
2	2	696	128.73	0.031	47
3	5	693	128.575	0.046	113
4	10	684	127.576	0.046	229
5	25	668	126.615	0.093	565
6	50	650	126.615	0.109	650
7	100	3	14.276	0.078	2342
8	150	2	21.164	0.062	2341
9	200	2	27.808	0.124	2341
10	300	2	41.525	0.140	2341

The set of reads contained 3817 reads all of which were derived from ‘Influenza A virus (A Puerto Rico 834 (H1N1)) segment 1, complete sequence’. Out of 3817 reads the method extracted 696 unique reads which were then used in the MDL proposed scheme. All the reference sequences were derived from the same Influenza A (H1N1) virus. Ref. Seq. 1% used in S.No. 1, has a length which is 1% of the actual genome. Similarly Ref. Seq. 25% has a length which is a quarter of the length of the actual genome. All other genomes were derived in a similar way. For, e.g., Ref. Seq. 200% has two H1N1 viruses concatenated together making the length twice that of the original H1N1 sequence. The code-length is calculated using Equation (3). The results show that the MDL proposed scheme chooses the best reference sequence, one which has the smallest code-length as determined by Equation (3). The MDL scheme does not choose smaller reference sequences with more unaligned reads rather than choosing larger reference sequence with smaller unaligned reads. The experiment also proves the correctness of the optimal reference sequence as it chooses Ref. Seq. 7, (shown underlined), since it has the smallest code-length, as the optimal reference sequence. It was Ref. Seq. 7 from which all the reads were derived from. Since the MDL scheme chooses Ref. Seq. 7 as the optimal sequence, the experiment also proves the correctness of the reference sequence chosen.

**Table 9:** The exeriment tests the proposed MDL scheme on a single set of reads yet on a number of reference sequences

**S.No.**	**Ref. Seq. (%)**	**No. of unaligned reads**	**Code-length (KB)**	**Length of new Seq.**
1	75	172	25.91	1755
2	85	148	25.10	1989
3	95	123	24.20	2223
4	100	109	23.62	2341
5	105	108	24.22	2458
6	115	107	25.50	2692
7	125	106	26.78	2926

The set of reads, 390 in total, were derived from ‘Influenza A virus (A Puerto Rico 834 (H1N1)) segment 1, complete sequence’ using the ART read simulator for NGS with read length 30, standard deviation 10, and mean fragment length of 100, [79]. Similarly the reference sequences were also derived from the same H1N1 virus. Ref. Seq. 75% used in S.No. 1, has a length which is 75% of the actual genome. Similarly Ref. Seq. 125% has a quarter of the actual genome concatenated with the complete H1N1 genome making the total length 125% of H1N1. All other genomes were derived in a similar way. The code-length is calculated using Equation (3). The results show that the MDL proposed scheme chooses the correct reference sequence, Ref. Seq. 100%, (shown underlined) even when all the contending sequences are closely related to one another in terms of their genome and length.

## Discussion

The MDL proposed scheme was tested using two-set of experiments. In the first set the robustness of the proposed scheme was tested using reference sequences, both real and simulated, having four types of mutations {Inversions, Insertions, Deletions, SNPs} compared to the novel genome. This was done with the help of a program called change_sequence. The program ‘change_sequence’ requires the user to input *Υ*_*m*_, the probability of mutation, in addition to the original sequence from which the reference sequences are being derived. It start by traversing along the length of the genome, and each time it arrives at a new base, a uniformly distributed random generator generates a number between 0 and 100. If the number generated is less than or equal to *Υ*_*m*_ a mutation is introduced. Once the decision to introduce a mutation is made, the choice of which mutation still needs to be made. This is done by rolling a biased four sided dice. Where each face of the dice represents a particular mutation, i.e., {inversion, deletion, insertion, and SNPs}. The percentage bias for each face of the dice is provided by the user as four additional inputs, *Υ*_inv_, for the percentage bias for inversions, *Υ*_indel_, representing percentage bias for insertions and deletions and *Υ*_SNP_ for SNPs. If the dice chooses inversion, insertion or deletion as a possible mutation it still needs to choose the length of the mutation. This requires one last input from the user, *Υ*_len_, identifying the upper threshold limit of the length of the mutation. A uniformly distributed random generator generates a number between 1 and *Υ*_len_, and the number generated corresponds to the length of the mutation.

The proposed MDL scheme is shown to work successfully, as it chooses the optimal reference sequence to be the one which has smaller number of SNPs, see Table 3, smaller number of insertions, see Table 4, and smaller number of deletions compared to the novel genome, see Table 5. The proposed MDL scheme is also seen to detect and rectify most, if not all, of the inversions present in the reference sequence, see Table 6. Since the code-length of *S*_*R*1_ is the same as *S*_*R*2_, and all the inversions of *S*_*R*2_ are rectified, the corrected *S*_*R*2_ sequence and *S*_*R*1_ sequence are equally good for reference assisted assembly.

The experiment carried out using Influenza viruses is shown in Table 7. One sequence was randomly chosen amongst the seven sequences and modified at random locations, using the same ‘change_sequence’ program, to form the novel sequence *S*_*N*_. The novel sequence contained {SNPs = 7, inversions = 4, deletions = 1, insertions = 3} as compared to the original sequence. The MDL process used the reads derived from *S*_*N*_ to compare seven sequences and determined Influenza virus B to be optimal reference sequence as it had the smallest code-length. The MDL process rectified all inversions while only one insertion was found. This meant that the remaining two insertions were smaller than *τ*_1_. The set of reads and Influenza virus B was then fed into MiB (**M**DL-**I**DITAP-**B**ayesian estimation comparative assembly pipeline) [80]. The MiB pipeline removes insertions and rectifies inversions using the MDL proposed scheme. IDITAP is a de-bruijn graph based denovo assembler that **I**dentifies the **D**eletions and **I**nserts them a**TA**ppropriate **P**laces. BECA (**B**ayesian **E**stimator **C**omparative **A**ssembler) helps in rectifying all the SNPs. The novel genome reconstructed by the MiB pipeline was one contiguous sequence with a length of 2368 bases and a completeness of 96.62%.

The second-set of experiment tests the correctness of the MDL proposed scheme, by testing the MDL scheme on a single set of reads but on a number of different reference sequences having a wide range of lengths. In the first test 3817 reads were derived from ‘Influenza A virus (H1N1) segment 1’ without any mutations, of which only 696 reads remained after collapsing duplicate reads. The reference sequences were also derived from the same H1N1 virus, with reference sequence (Ref. Seq.) 1% having a length which is 1% of the actual genome. Similarly Ref. Seq. 25% has a length which is a quarter of the length of the actual genome. Similarly Ref. Seq. 125% has a quarter of the actual genome concatenated with the complete H1N1 genome making the total length 125% of H1N1. All other reference sequences were derived in a similar way, see Table 8. The unique set of reads and the reference sequences were tested using the MDL proposed scheme, where the code-length was calculated using Equation (3). The results show that the MDL scheme does not choose smaller reference sequences with more unaligned reads rather it chooses the correct reference sequence, Ref. Seq. 7. It was Ref. Seq. 7 from which all the reads were derived from. Since the MDL scheme chooses Ref. Seq. 7 as the optimal sequence, this experiment further proves the correctness of the reference sequence chosen.

Lastly, the above experiment was repeated using a single set of reads derived from the same H1N1 virus segment 1, but this time containing mutations. The set of reads, 390 in total, were derived using the ART read simulator for NGS with read length 30, standard deviation 10, and mean fragment length of 100, [PUT ART Reference], see Table 9. The results show that the MDL proposed scheme chooses the correct reference sequence, Ref. Seq. 100%, even when all the contending reference sequences are closely related to one another in terms of their genome and length.

All simulations were carried out on Intel Core i5 CPU M430 @ 2.27 GHz, 4 GB RAM. Execution time of MDL proposed scheme have been provided in Table 8.

## Conclusions

The article explored the application of Two-Part MDL qualitatively and the application of Sophisticated MDL both qualitatively and quantitatively for selection of the optimal reference sequence for comparatively assembly. The article compared the MDL scheme with the standard method of “counting the number of reads that align to the reference sequence” and found that the standard method is not sufficient for finding the optimal sequence. Therefore, the proposed MDL scheme encompassed within itself the standard method of ‘counting the number of reads’ by defining it in an inverted fashion as ‘counting the number of reads that did not align to the reference sequence’ and identified it as the ‘data given the hypothesis’. Furthermore, the proposed scheme included the model, i.e., the reference sequence, and identified the parameters $\left(\theta_{M_{i}}\right)$ for the model (*M*_*i*_) by flagging each base of the reference sequence with {−1, 0, 1}. The parameters of the model helped in identifying inversions and thereafter rectifying them. It also identified locations of insertions. Insertions larger than a user defined threshold *τ*_1_ and smaller than *τ*_2_ were removed. Therefore, the proposed MDL scheme not only chooses the optimal reference sequence but also fine-tunes the chosen sequence for a better assembly of the novel genome.

Experiments conducted to test the robustness and correctness of the MDL proposed scheme, both on real and simulated data proved to be successful.

## Competing Interests

The authors declare that they have no competing interests.
